# Prosthetic joint infection after total hip arthroplasty caused by *Sneathia sanguinegens*: A case report (CARE-complaint)

**DOI:** 10.1097/MD.0000000000022494

**Published:** 2020-10-09

**Authors:** Shohei Kawakami, Ken Iwata, Masashi Shimamura, Tasuku Mashiba, Kyoko Yokota, Kiyoshi Negayama, Kiyofumi Ohkusu, Tetsuji Yamamoto

**Affiliations:** a Department of Orthopedic Surgery, Faculty of Medicine, Kagawa University, Miki-cho, Kita-gun; b Department of Internal Medicine, Kagawa Prefectural Central Hospital, Takamatsu City; c Department of Infectious Diseases, Kagawa University Hospital, Miki-cho, Kita-gun, Kagawa; d Department of Microbiology, Tokyo Medical University, Shinjuku-ku, Tokyo, Japan.

**Keywords:** case report, polymerase chain reaction, prosthetic joint infection, *Sneathia sanguinegens*, total hip arthroplasty

## Abstract

**Introduction:**

*Sneathia sanguinegens***(***S sanguinegens)* is a gram-negative rod-shaped bacterium mostly reported to cause a perinatal infection, and there are no reports of *S sanguinegens* in prosthetic joint infection (PJI). The purpose of this report is to describe a very rare case of PJI after total hip arthroplasty (THA) caused by *S sanguinegens*.

**Patient concerns:**

A 79-year-old woman presented with right coxalgia, inability to walk, and a fever of 39°C. She had undergone THA 28 years earlier for osteoarthritis of the hip.

**Diagnoses:**

The diagnosis was acute late-onset PJI, because blood tests revealed marked inflammatory reaction and computed tomography showed an abscess at the right hip joint; synovial fluid analysis resulted in detection of a gram-negative bacillus.

**Intervention:**

Surgical debridement with retention of the implant and antibiotic therapy was performed.

**Outcomes:**

One month after surgery, polymerase chain reaction (PCR) assay showed that the pathogen was 99.9% likely to be *S sanguinegens*. There has been no recurrence of infection or loosening of the implant in the 2 years since her surgery.

**Lessons:**

PCR should facilitate detection of previously unknown pathogens and potentially novel bacterial species.

## 1 Introduction

Total hip arthroplasty (THA) improves quality of life in patients with osteoarthritis or other diseases of the hip. However, patients who undergo this procedure are at risk of prosthetic joint infection (PJI), which requires surgery and long-term antibiotic therapy. Although prophylactic antibiotic therapy is recommended to prevent PJI,^[1]^ 0.4% to 1.4% of patients have been reported to develop the condition within 1 year of THA.^[2,3]^ Here, we report the rare case of a patient who developed anaerobic PJI caused by *Sneathia sanguinegens (S sanguinegens)*. This organism is known to cause infection in obstetric patients^[4–6]^ but, to our knowledge, has never been reported in patients who have undergone PJI. This study was approved by the IRB of the author's affiliated institution (the study number, 29-213). The patient and her family provided informed consent for data from the case to be published.

## 2 Case report

**Figure 1:**
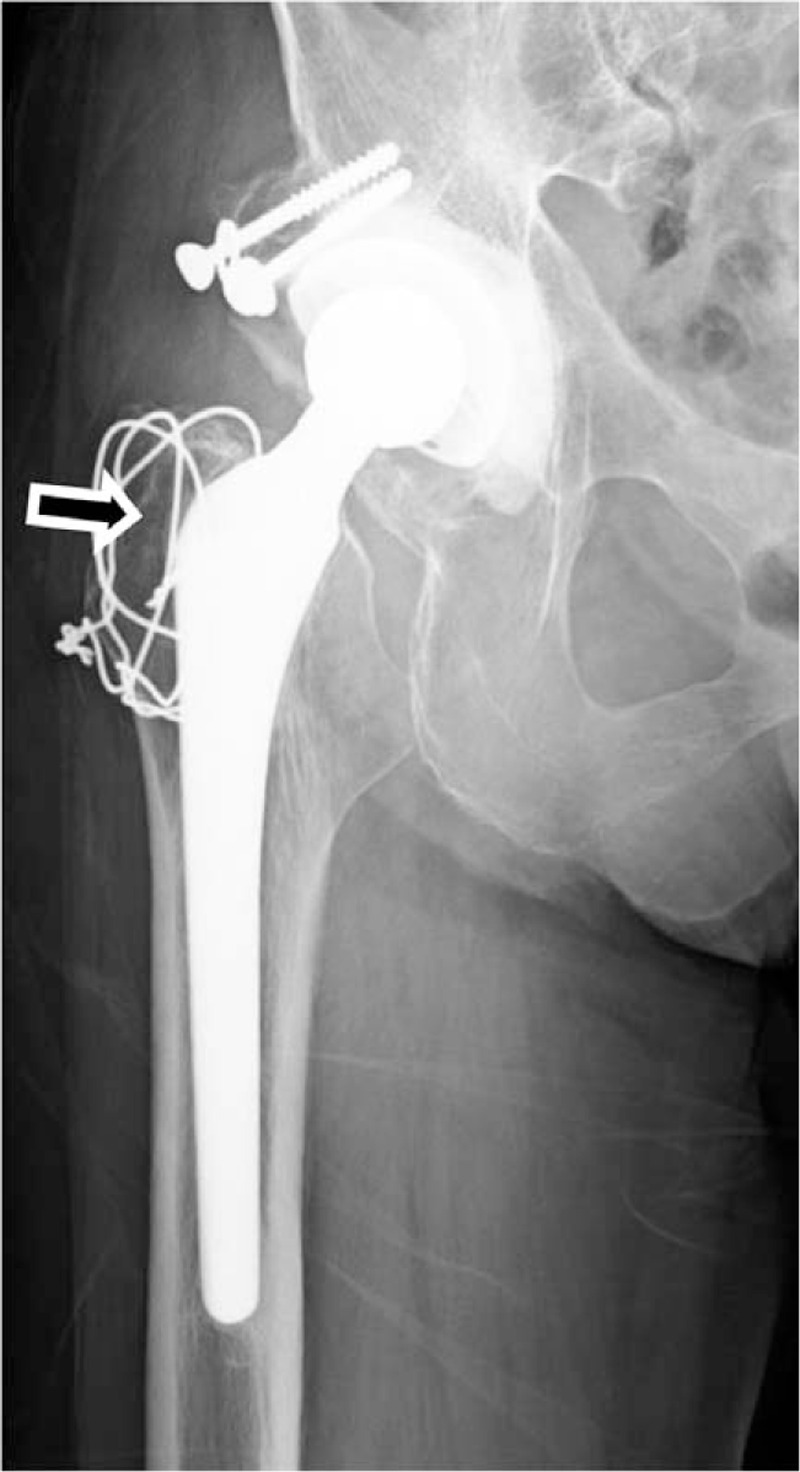
Radiological images. Plain radiograph of the right hip at the time of admission showing slight osteolytic changes at around the implant (black arrow), but no evidence of loosening of the implant.

**Figure 2:**
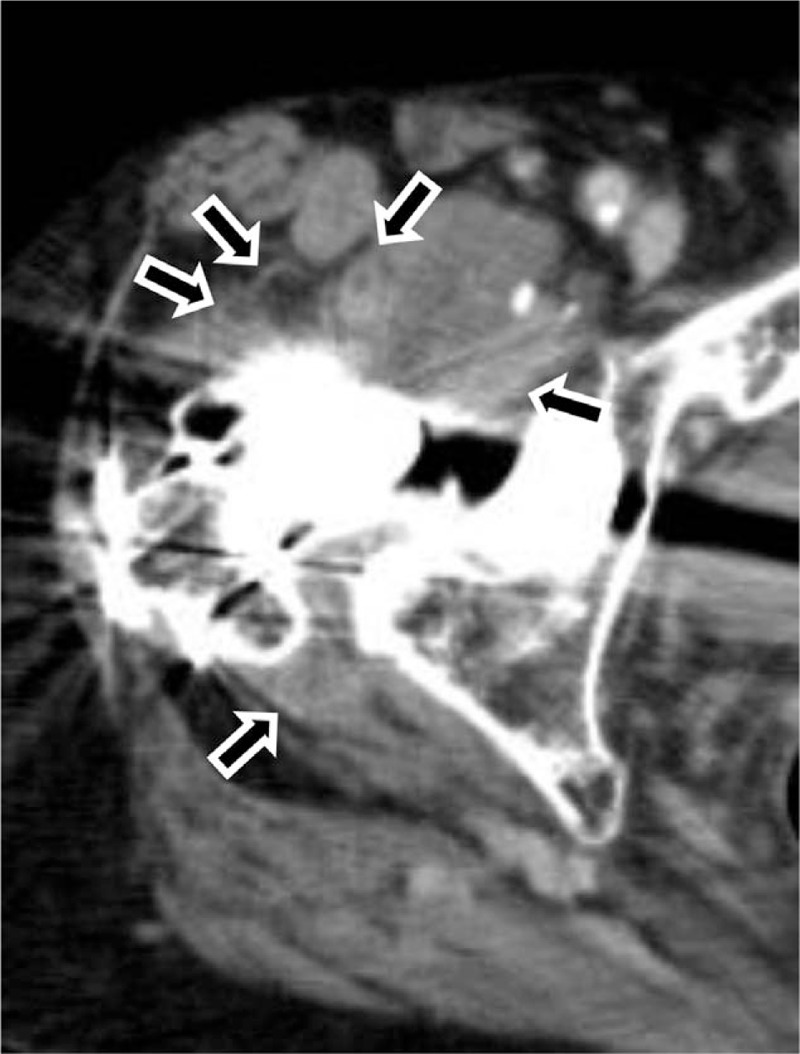
Radiological images. Contrast-enhanced computed tomography scan showing a ring-enhancing region around the right hip joint suggesting abscess formation (black arrow).

**Figure 3:**
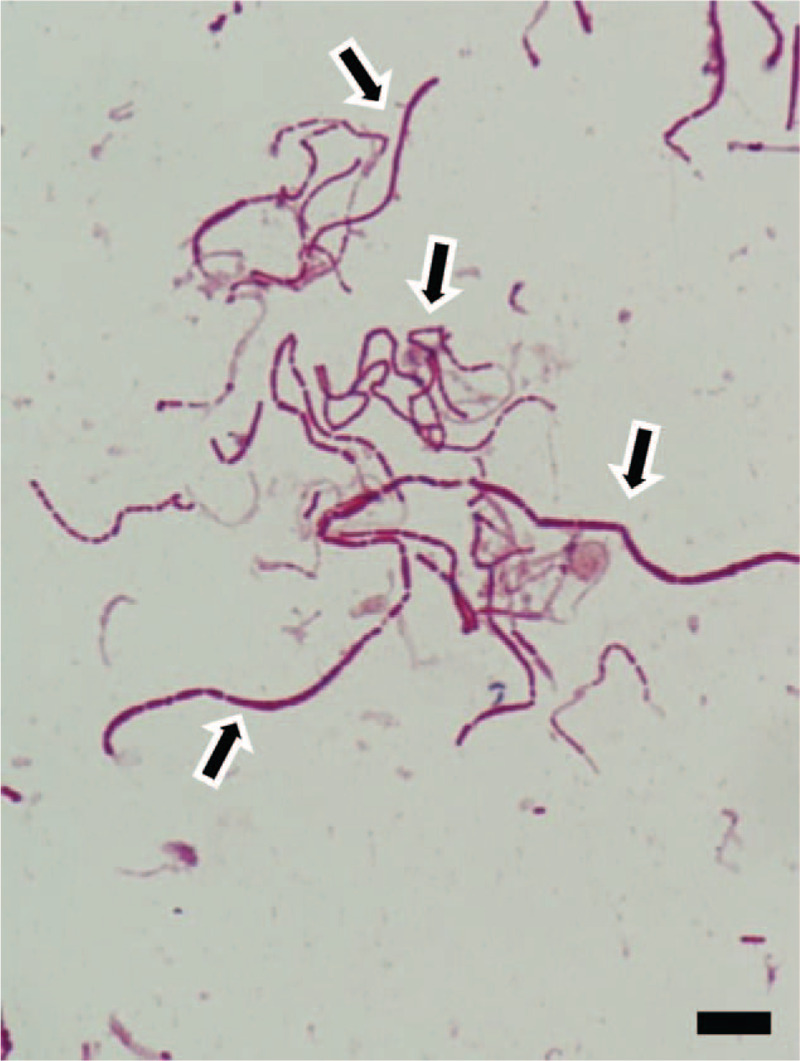
Photograph of Gram staining of synovial fluid of hip joint. Analysis reveals a gram-negative bacillus (black arrow). Scale bar, 10 μm.

**Figure 4:**
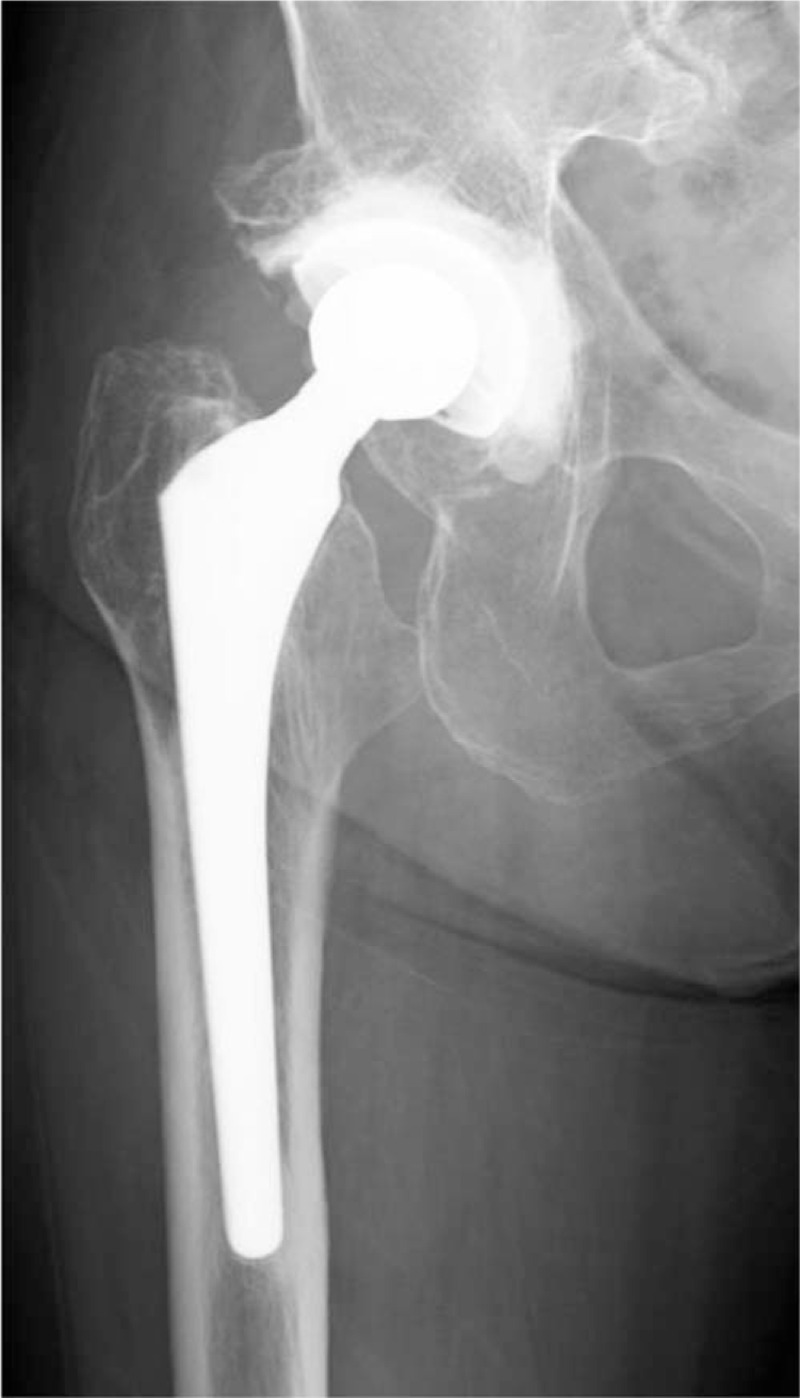
Plain radiograph immediately after surgery. The implant is retained, the wire and screw were removed.

**Figure 5:**
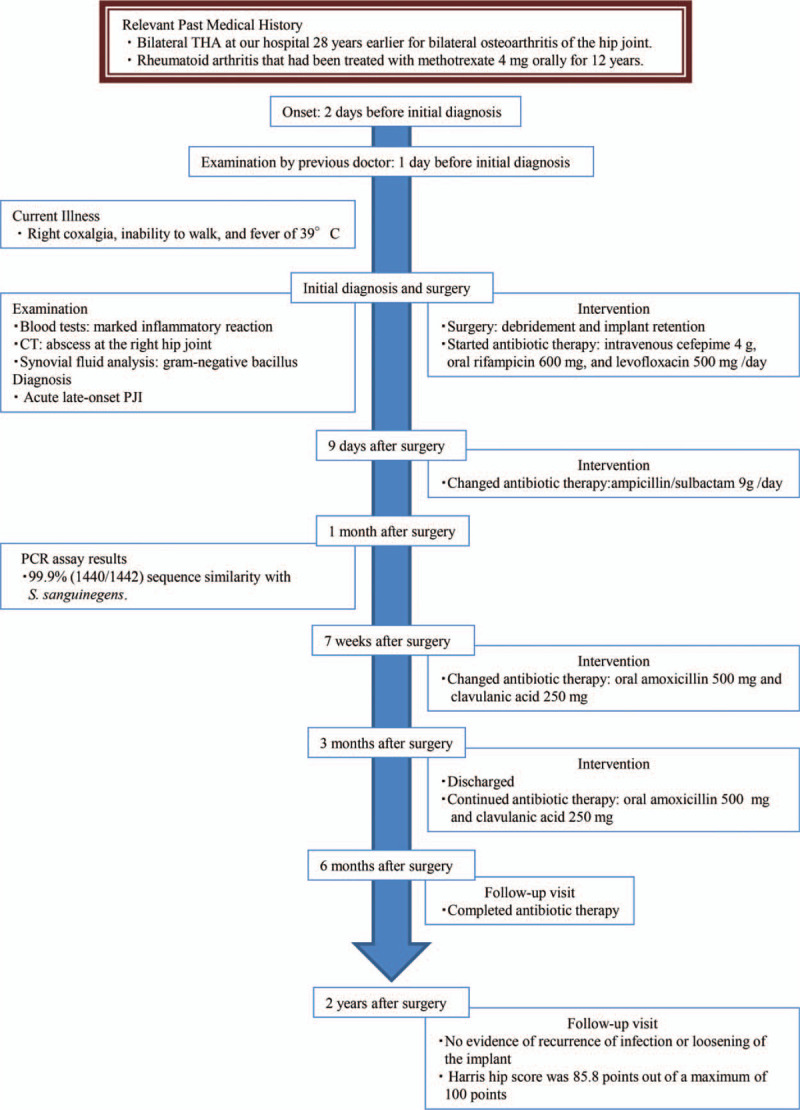
Timeline. Historical and current information from this episode of care.

A 79-year-old woman was referred to us by another hospital with right hip pain, inability to walk, and a fever of 39°C. She began to feel right coxalgia 2 days before and visited the previous hospital 1 day before. She had undergone bilateral THA at our hospital 28 years earlier for bilateral osteoarthritis of the hip joint. Her past medical history included rheumatoid arthritis that had been treated with methotrexate 4 mg orally for 12 years. She had a C-reactive protein level of 228.4 mg L^−1^ and a white blood cell count of 17,700 μL^−1^, indicating an inflammatory state. Blood culture was negative. Range of motion at the right hip joint was limited. Plain radiography of the right hip at the time of admission showed a slight peri-implant osteolytic change (Fig. 1, black arrow), but no evidence of loosening of the implant. Contrast-enhanced computed tomography showed a ring-enhancing region suggestive of abscess formation around the right hip joint (Fig. 2, black arrow). Synovial fluid analysis resulted in detection of a gram-negative bacillus (Fig. 3, black arrow). PJI was suspected and the plan was to treat with surgical debridement, antibiotic therapy, with retention of the implant. Surgery was performed via the previous incision using a posterolateral approach. Although pus and synovial inflammation were found in the right hip joint, we considered that the implant could be retained because there was no loosening of the femoral stem, the acetabular cup was stable, and the polyethylene liner showed an acceptable level of wear. After debridement of infectious soft tissue, the wire and screw were removed and sufficient lavage was performed (Fig. 4). A gram-negative bacillus was detected in the synovial fluid intraoperatively, so antibiotic therapy (intravenous cefepime 4 g and oral rifampicin 600 mg and levofloxacin 500 mg per day) was started. Nine days after surgery, at which time it was confirmed that the causative pathogen was an anaerobe, the patient was switched from cefepime to ampicillin/sulbactam 9 g per day for 6 weeks. One month after surgery, this isolate was identified by sequencing 1400 bp of the 16S rRNA gene after amplification by polymerase chain reaction (PCR). The isolate showed 99.9% (1440/1442) sequence similarity with *S sanguinegens*. After administration of ampicillin/sulbactam, the patient was switched to oral amoxicillin 500 mg and clavulanic acid 250 mg per day. At 3 months after surgery, she was discharged and continued on the same oral antibiotics for 3 months. At the most recent follow-up 2 years after surgery, she was able to walk without pain. There was no evidence of recurrence of infection or loosening of the implant, and Harris hip score was 85.8 points out of a maximum of 100 points.^[7]^ The timeline for this case is summarized in Figure 5.

## 3 Discussion

*S sanguinegens* is an anaerobic bacterium that is part of the normal flora of the oral cavity, gastrointestinal tract, and female genital tract. It is a nonmotile, non-spore-forming, rod-shaped gram-negative organism that requires complex nutrients and develops into small colonies when incubated on blood agar plates for several days at 37°C under anaerobic conditions.^[6]^*S sanguinegens* is mostly reported in the obstetric setting, including in mothers postpartum and neonates.^[4–6]^ Hanff et al originally named this organism *Leptotrichia sanguinegens*; however, Collins et al^[5]^ renamed it *S sanguinegens* on the basis of phenotypic and phylogenetic evidence. There has been a report of *S sanguinegens* as the cause of infective endocarditis in a prosthetic valve endocarditis;^[8]^ however, to our knowledge, the present case is the first report of *S sanguinegens* as the causative pathogen in PJI.

Our patient was an elderly postmenopausal woman whose immunocompetence may have been compromised by treatment for rheumatoid arthritis. She had had no episodes of bacteremia or infectious events in the 28 years since bilateral THA, suggesting a hematogenous infection. *S sanguinegens* was identified by cloning and sequencing of the 16S rRNA gene,^[6]^ and in this case there was 99.9% similarity to the bacterial sequences deposited in GenBank. Beta-lactam antibiotics are generally effective for anaerobic infection, and intravenous administration of ampicillin/sulbactam with oral rifampicin achieved a good outcome in our patient. De Martino et al^[6]^ also reported good results for treating *S sanguinegens* infection with amoxicillin and/or clavulanic acid.

It is unknown which of the available treatments, namely, debridement, antibiotics, and implant retention (DAIR) and 1-stage or 2-stage revision, achieves the best functional outcomes in patients with PJI at the hip.^[9–11]^ Grammatopoulos et al^[11]^ reported that the outcomes were significantly better if DAIR was performed within 7 days of onset of symptoms. In our patient, the onset of symptoms developed over a short period and intraoperative findings confirmed that the stability of the implant was not compromised. Furthermore, the tools needed to remove the implant were not immediately available because her implants were of an older type; therefore, we performed DAIR to control local infection and there was no recurrence. Treatment with debridement and appropriate antibiotics achieved a successful outcome, consistent with the report by Grammatopoulos et al.^[11]^ There has been a report of PJI caused by an unidentified anaerobe, but whether or not the species was Sneathia was not clinically important because the infection was treated successfully using broad-spectrum anaerobic antibiotics.

PCR should facilitate detect previously unknown pathogens and potentially novel bacterial species.^[12,13]^ Fenolla et al^[14]^ proposed use of the 16S rRNA gene PCR assay for culture-negative cases when infection is suspected on the basis of clinical signs and symptoms or if inflammation is present. Even if synovial fluid culture is negative, PCR is necessary because late PJI can be caused by slow-growing bacteria, such as *S sanguinegens*.

## Author contributions

**Investigation:** Masashi Shimamura, Tasuku Mashiba, Kyoko Yokota, Kiyoshi Negayama, Kiyofumi Ohkusu, Tetsuji Yamamoto.

**Writing – original draft:** Shohei Kawakami.

**Writing – review & editing:** Ken Iwata.

## Abbreviations

Abbreviations: DAIR = debridement, antibiotics and implant retention, PCR = polymerase chain reaction, PJI = prosthetic joint infection, THA = total hip arthroplasty.

## References

[1] AlBuhairan B Hind D Hutchinson A. Antibiotic prophylaxis for wound infections in total joint arthroplasty. J Bone Joint Surg 2008;90:915–9. 18591602 10.1302/0301-620X.90B7.20498

[2] Biau DJ Leclerc P Marmor S. Monitoring the one year postoperative infection rate after primary total hip replacement. Int Orthop 2012;36:1155–61. 22207406 10.1007/s00264-011-1444-y PMC3353069

[3] Triantafyllopoulos GK Soranoglou VG Memtsoudis SG. Rate and risk factors for periprosthetic joint infection among 36494 primary total hip arthroplasties. J Arthroplasty 2018;33:1166–70. 29248486 10.1016/j.arth.2017.11.040

[4] Hanff PA Rosol-Donoghue JA Spiegel CA. Leptotrichia sanguinegens sp. nov., a new agent of postpartum and neonatal bacteremia. Clin Infect Dis 1995;20: (suppl 2): S237–9. 7548563 10.1093/clinids/20.supplement_2.s237

[5] Collins MD Hoyles L Tornqvist E. Characterization of some strains from human clinical sources which resemble “Leptotrichia sanguinegens”: description of Sneathia sanguinegens sp. nov., gen. nov. Syst Appl Microbiol 2001;24:358–61. 11822670 10.1078/0723-2020-00047

[6] De Martino S Mahoudeau I Brettes JP. Peripartum bacteremias due to Leptotrichia amnonii and Sneathi sanguinegens, rare causes of fever during and after delivery. J Clin Microbiol 2005;42:5940–3. 10.1128/JCM.42.12.5940-5943.2004 PMC535221 15583348

[7] Harris WH. Traumatic arthritis of the hip after dislocation and acetabular fractures: treatment by mold arthroplasty. An end-result study using a new method of result evaluation. J Bone Joint Surg Am 1969;51:737–55. 5783851

[8] Kotaskova I Nemec P Vanerkova M. First report of Sneathia sanguinegens together with Mycoplasma hominis in postpartum prosthetic valve infective endocarditis: a case report. BMC Infect Dis 2017;17:563. 28806998 10.1186/s12879-017-2654-8 PMC5557263

[9] Klouche S Leonard P Zeller V. Infected total hip arthroplasty revision: one- or two-stage procedure? Orthop Traumatol Surg Res 2012;98:144–50. 22364829 10.1016/j.otsr.2011.08.018

[10] Zimmerli W Trampuz A Ochsner PE. Prosthetic-joint infections. N Engl J Med 2004;351:1645–54. 15483283 10.1056/NEJMra040181

[11] Grammatopoulos G Bolduc ME Atkins BL. Functional outcome of debridement, antibiotics and implant retention in periprosthetic joint infection involving the hip. Bone Joint J 2017;99-B:614–22. 28455470 10.1302/0301-620X.99B5.BJJ-2016-0562.R2

[12] Omar M Petri M Hawi N. Higher sensitivity of swab polymerase chain reaction compared with tissue cultures for diagnosing periprosthteic joint infection. J Orthop Surg (Hong Kong) 2018;26:1–5. 10.1177/2309499018765296 29540099

[13] Palan J Nolan C Sarantos K. Culture-negative periprosthetic joint infections. Gen Orthop 2019;4:585–94. 10.1302/2058-5241.4.180067 PMC6836077 31754464

[14] Fenollar F Roux V Stein A. Analysis of 525 samples to determine the usefulness of PCR amplification and sequencing of the 16S rRNA gene for diagnosis of bone and joint infections. J Clin Microbiol 2006;44:1018–28. 16517890 10.1128/JCM.44.3.1018-1028.2006 PMC1393109

